# A gene signature associated with PTEN activation defines good prognosis intermediate risk prostate cancer cases

**DOI:** 10.1002/cjp2.94

**Published:** 2018-02-28

**Authors:** Chee W Ong, Pamela Maxwell, Muhammad A Alvi, Stephen McQuaid, David Waugh, Ian Mills, Manuel Salto‐Tellez

**Affiliations:** ^1^ Movember FASTMAN Centre of Excellence, Centre for Cancer Research and Cell Biology, Queen's University Belfast Northern Ireland, UK

**Keywords:** prostate cancer, prognostic, gene signature

## Abstract

Accurate identification of intermediate risk (Gleason 3 + 4 = 7) prostate cancer patients with low risk of disease progression is an unmet challenge in treatment decision making. Here we describe a gene signature that could guide clinicians in the selection of patients with intermediate stage clinically localized prostate cancer for active surveillance. We examined six major drivers of aggressive disease – PTEN, MYC, RB1, TP53, AURKA, AR – by immunohistochemistry in a focused (N = 69) cohort predominantly consisting of intermediate risk prostate cancer. Fuzzy clustering and unsupervised hierarchical clustering were utilized to determine the correlation of gene expression and methylation values with immunohistochemical expression. From the immunohistochemistry observation, we found that intermediate risk prostate cancer cases could be classified as ‘complex’ (differential expression of more than one driver) or ‘simple’ (differential expression of only one). Focussing on the ‘simple’ cases, expression and methylation profiling generated signatures which correlated tightly only with differential PTEN expression and not with any of the other drivers assessed by immunohistochemistry. From this, we derived a geneset of 35 genes linked to high PTEN expression. Subsequently we determined its prognostic significance in intermediate‐risk cases extracted from three publicly available clinical datasets (Total N = 215). Hence, this study shows that, by using immunohistochemistry as an upfront stratifier of intermediate risk prostate cancers, it is possible to identify through differential gene expression profiling a geneset with prognostic power across multiple cohorts. This strategy has not been used previously and the signature has the potential to impact on treatment decisions in patients for whom decision making is currently empirical at best.

## Introduction

Prostate cancer remains the second most common cancer‐related death for men in developed nations 1. In the United Kingdom, prostate cancer has become the most common form of cancer in males, surpassing lung and bowel cancers 2. This is attributed to population aging in the region and the prevalent use of prostate‐specific antigen as a screening biomarker 3. Despite increased understanding of prostate cancer biology, the current standard of care is still dependent on the Gleason score of the patient's tumour 4. In general, most prostate tumours are low and intermediate grades (Gleason score 6 and 7). To prevent overtreatment, there has been an emerging approach of non‐treatment or active surveillance for patients who present with tumours graded lower than Gleason score 7. Here the intent to prevent overtreatment could obscure the opportunity for early intervention, especially in potentially fast‐growing and aggressive tumours. Thus, molecular genomic analysis may be useful in identifying such potentially aggressive tumours that cannot be distinguished by existing practices.

Several studies have utilized next‐generation high‐throughput methodologies to characterize the different molecular genomic aberration events in prostate cancer. These studies have collectively shown that the most frequent genomic events in prostate cancer are the amplification or mutation of the androgen receptor, loss of the *PTEN* tumour suppressor gene, and the genomic rearrangement events surrounding the oncogenic transcription factor *ERG*. Particularly, loss of *PTEN* was associated with advanced stage and poor prognosis in prostate cancer 5, 6, 7, 8, 9. These studies have allowed researchers to understand each major genomic aberration involved in prostate cancer development and progression.

Despite understanding the various genomic events in prostate cancer, the molecular characterization of cancer subtypes by genomic analysis is more successful and widespread in other cancer types, such as breast and colorectal cancers 10, 11, 12, 13. It is only in recent years that genomic profiling has been employed in a therapeutic setting in prostate cancer, giving rise to a number of commercially available transcript signatures that are now being used as clinical nomograms to predict disease outcome (Prolaris, Oncotype DX Genomic Prostate Score, and Decipher) 14, 15, 16. All three assays are primarily designed and used in the setting of diagnostic biopsies for advanced prostate cancers, but occasionally are also used in intermediate risk prostate cancer. The Prolaris genomic assay (Myriad Genetics, UT, USA) is a 46‐gene expression panel that encompasses mostly cell‐cycle progression genes that improves prediction of metastatic progression risk in men undergoing external beam radiation after radical prostatectomy 14. Similarly, the 22‐gene Decipher genetic test (GenomeDX Biosciences, Vancouver, Canada) 16 is reported to enhance prediction of metastatic progression risk in men undergoing External Beam Radiation Therapy 17 as well as prostate cancer‐specific mortality 18. Conversely, the 17‐gene Oncotype DX Genomic Prostate Score assay (Genomic Health Inc., CA, USA) 15 is reported to be significantly associated with adverse pathological features as well as time to metastasis 19. Except for one common gene between the Prolaris and Decipher assay, there are no overlapping genes reported between these three gene‐expression‐based tests. These tests correlate to some degree with high grade, high stage disease. Thus, in‐depth molecular characterisation of the most demanding clinical risk group for prostate cancer, intermediate risk disease, may aid in the further stratification of patients. This can determine who might benefit from early treatment intervention provided that it is possible to subdivide these cases in some manner prior to transcript or methylation profiling.

Here, we systematically profiled a small cohort of radical prostatectomy cases consisting almost entirely of intermediate risk, Gleason 7 (3 + 4), disease. Using an initial immunohistochemical analysis of defined genetic markers, followed by subsequent gene expression, and methylation profiling of the samples, we have integrated this data to derive a 35‐geneset which is prognostic in single grade disease across multiple cohorts.

## Patients and methods

### Patient datasets

**Figure 1:**
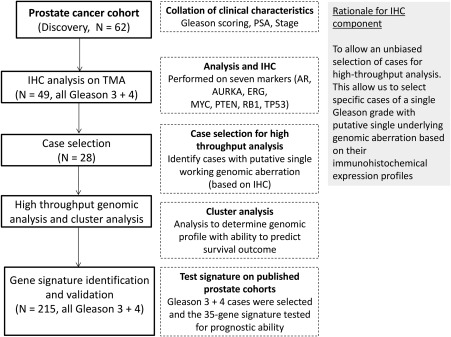
Flowchart outlining the workflow and patient selection for determining the prognostic ability of the gene signature in Gleason 7 (3 + 4) patients.

**Figure 2:**
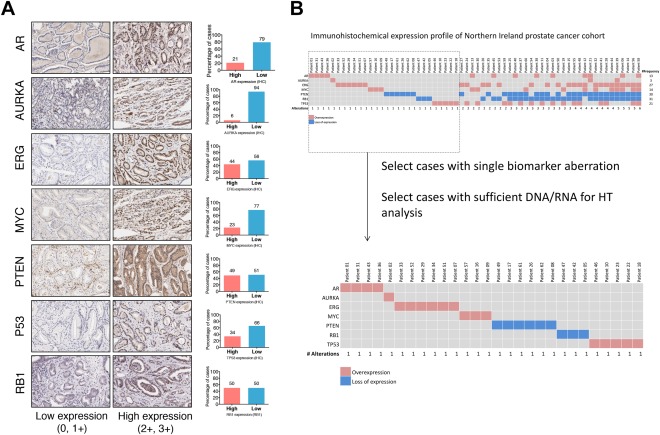
(A) Representative immunohistochemical expression of the markers utilized in the selection of cases for high‐throughput analysis. Bar charts adjacent to each marker describe the dichotomized expression frequencies (0, 1+ versus 2+, 3+) of the corresponding marker. (B) Diagram describing the sample selection criteria for high‐throughput analysis. The patients (N = 62) were aligned according to the number of genomic aberrations present, in ascending order. A total of 28 cases were chosen on the basis that they each represent a single biomarker aberration based on their immunohistochemical expression. HT, high throughput.

For this study, the main clinical cohort is referred to as the Northern Ireland dataset (N = 62). Ethical permission for the study was granted by the Northern Ireland Biobank (Ethics: 11/NI/0013/NIB13–0074). The clinical features of this cohort are described in supplementary material, Table S1. For validation of the gene expression signature, the datasets used are referred as the Taylor dataset (GSE21032), the Sboner dataset (GSE16560), and the Gulzar dataset (GSE40272) 20, 21, 22. All selected cases were assigned a combined Gleason score of 3 + 4. Gleason 4 + 3 cases were excluded. Both Taylor and Gulzar cohorts were radical prostatectomy specimens. The Sboner cohort comprised transurethral resection specimens. Overall survival was used as the outcome measure for the Taylor cohort. Recurrence free survival was used as the outcome measure for the Gulzar and Sboner cohort (supplementary material, Table S2). A total of 28 cases from the Northern Ireland dataset were analysed for gene expression, methylation, and mutations (Figure 1). These cases were selected based on immunohistochemical expression and belonged to the ‘simple’ subgroup (or cases associated with abnormal expression of only one marker) as outlined in Figure 2.

### Patient specimens and nucleic acid extraction

Formalin‐fixed and paraffin‐embedded (FFPE) radical prostatectomy tissue specimens were obtained from the Northern Ireland Biobank constituting the NI Dataset. Sequential 4 μm sections of each case were sectioned and placed on glass slides (a total of 20 μm). Tumour regions were macro‐dissected into sterile 1.5 ml Eppendorf tubes. RNA was extracted using the Qiagen RNAeasy kit (Qiagen GmbH, Hilden, Germany). Genomic DNA was extracted using the Promega Genomic DNA extraction kit (Promega, WI, USA). All standard procedures were in accordance with the manufacturer's protocols. Extracted DNA and RNA were quantified and assessed for their quality using Agilent Bioanalyser chips (Agilent Technologies, CA, USA).

### Tissue microarray (TMA) construction and immunohistochemistry (IHC)

For the construction of tissue microarrays, representative FFPE tissues from selected resection materials were cored (0.6 mm) and arrayed into a donor recipient block using a tissue microarrayer (Beecher Instruments, WI, USA). Consecutive TMA paraffin sections of 4 μm thickness were cut and placed onto silanated slides for immunohistochemical detection.

Immunohistochemistry was performed for AR, AURKA, ERG, MTOR, P53, PTEN, and RB1. Standard processing steps for each antibody were in accordance with manufacturer's instructions. In brief, heat‐induced antigen retrieval with epitope retrieval ER1 solution (Leica Biosystems, Newcastle, UK) was performed for 20 min prior to incubation with primary antibody. Slides were incubated with primary antibody at optimized concentration. After incubation, slides were washed with Bond washing buffer (Leica Biosystems) and incubated with secondary antibody (Bond Polymer Refine kit; Leica Biosystems). Subsequently chromogenic detection was achieved by incubation with 3,3′‐diaminobenzidine (DAB) followed by Bond DAB enhancer (Leica Biosystems). All slides were counterstained with haematoxylin and dehydrated through ascending ethanol to xylene before mounting. Further details regarding the use of each commercially available antibody including the dilution used are described in supplementary material, Table S3.

### Scoring criteria for immunohistochemistry expression

A modified Allred scoring method was utilized for evaluating the immunohistochemical expression of each marker 23. First, intensity and proportion of positive immunoreactivity of each marker were evaluated in each core without knowledge of the clinicopathological information. The staining intensity of each marker was scored as 0 (negative), 1+ (weak), 2+ (moderate), and 3+ (strong) while the proportion of positive immunoreactivity was scored in percentages. Subsequently, a dichotomous expression score was obtained by considering both the staining intensity and the proportion of stained cells within each core (supplementary material, Table S4). In brief, the intensity was scored as ‘low expressing’ (no or low staining in less than 50% of cells) or ‘high expressing’ (moderate or intense staining in more than 50% of cells). The majority expression scores were then taken as the overall score. Each TMA was analysed and scored by three observers independently (CWO, SM, and MST).

In view of the importance of PTEN after the initial analysis of results, a thorough validation of PTEN analysis at all levels was carried out in parallel while delivering the results of this study 24.

### Whole genome gene expression

Whole genome gene expression analysis was performed using the Illumina (Illumina, CA, USA) WG‐DASL assay in accordance with the manufacturer's protocol. In brief, 100 ng of FFPE RNA was converted to cDNA by the WG‐DASL assay using biotin‐tagged random nonamer and oligo (dT) primers. The biotinylated cDNA was then mounted onto a streptavidin‐coated support and further extended and ligated by gene‐specific oligonucleotides. Subsequently, polymerase chain reaction (PCR) amplification was performed. The resulting PCR products were eluted and hybridized to the Illumina Human‐Ref v3.0 Beadchip and scanned with the Illumina iScan Reader (Illumina). The image intensity values from the microarray images generated were then analyzed using the GenomeStudio Gene Expression Module (Illumina) software. The processed expression values were subsequently used for further analysis in this study.

### Whole genome methylation

Whole genome methylation analysis was performed using the Illumina Infinium HD (Illumina) assay in accordance with the manufacture's protocol. In brief, 1000 ng of genomic DNA extracted from the FFPE samples was firstly treated with sodium bisulphite to convert unmethylated cytosines to uracils. The bisulphite‐treated DNA was denatured and isothermally amplified overnight. After amplification, post‐amplified DNA was fragmented using a proprietary enzymatic process and precipitated using isopropanol. Precipitated DNA was collected by centrifugation and re‐suspended in a hybridization buffer, prior to hybridisation onto the Infinium 450K Beadchip (Illumina). The loaded chip underwent further extension and staining steps. Subsequently, the Illumina iScan reader was used to derive image intensity values from the stained chip using the high‐resolution scans of the chip. The image intensity values were processed and normalized using the GenomeStudio Gene Expression Module (Illumina). The processed methylation values were subsequently used for further analysis in this study.

### Cancer gene‐targeted next‐generation DNA sequencing

The Ion Ampliseq (Life Technologies, Carlsbad, CA, USA) assay simultaneously amplified 50 oncogenes and tumour suppressor genes covering 2800 COSMIC mutations in a single‐tube reaction. A minimum of 50 ng of FFPE DNA was used for molecular profiling according to the manufacturer's instructions with the Ion PGM system. In brief, the pooled DNA was paired and amplified with Ion Torrent adapters to produce a DNA template library. The resulting library then underwent sample emulsion PCR in which copies of the DNA template were allowed to amplify in the Ion Sphere Particles (ISP). Subsequently, the ISPs were recovered and barcoded. Next, barcoded samples were sequenced on the Ion Torrent PGM for 65 cycles, as per the recommended protocol. Finally, the resulting data were analysed for single nucleotide polymorphism by the proprietary Variant Caller Plugin within the Ion Torrent software suite (Life Technologies).

### Cluster analysis of gene expression and methylation data

For the 28 cases interrogated from the Northern Ireland dataset, raw intensity data from gene expression and methylation data were exported from GenomeStudio software (Illumina) and Log2 transformed for cluster analysis. The nonnegative matrix factorisation (NMF) package for the R statistical software was used for the cluster analysis of the whole genome gene expression and methylation values 25. The NMF method allowed identification of clusters in an unsupervised manner based on the Euclidean distance and average linkage. This was performed over genes with highest variation across patients.

### Risk stratification analysis

To assess the prognostic performance of the gene signature after identification, a prognostic index based on SurvExpress was used 26. This prognostic index is the linear component of the exponential function used to measure the level of association of the gene signature in a Cox proportional hazards model. More specifically, the prognostic index can be calculated as: PI  =  *β*_1_ *χ*_1_+  *β*_2_ *χ*_2_+… +  *β*_p_ *χ*_p_ in which *χ*_1_ refers to the expression value of Gene_1_ and *β*_i_ refers to the risk coefficient obtained from the Cox modelling. The median value (PI = −85.4) of the prognostic index for the training cohort (GSE21032) was then used to stratify risk groups. In subsequent analysis, high risk cases were cases with a prognostic index above the determined median value. Gene expression data and associated clinical parameters deposited at Gene Expression Omnibus were downloaded for GSE21032, GSE16560, and GSE40272 [20–22] and evaluated using the SurvExpress prognostic index.

### Survival analysis

For graphical representation of the difference in survival outcome between risk groups for each dataset, the Kaplan–Meier method was used with differences assessed by the log‐rank method. 10‐year recurrence‐free survival (any recurrence, or death from any cause, were considered as events) and overall survival (death from any cause) were used as endpoints. Classification of risk was classified according to groups assigned using the SurvExpress prognostic index. All survival data were analysed and graphically presented using GraphPad Prism 6 (Graphpad Software, CA, USA) with the level of statistical signature set at *p* < 0.05.

## Results

In consideration of the heterogeneous nature of prostate cancer, we selected cases from our cohort that were associated with a potential underlying genomic aberration. Using immunohistochemistry as a preliminary approach to select for such cases, we selected a panel of immunohistochemical markers (as described in Figure 1). These markers, PTEN, MYC, RB1, TP53, AURKA, AR, are all drivers of aggressive or advanced disease with associated therapeutics in development which could aid treatment 27. Upon scoring the immunohistochemical expression using modified Allred scoring criteria, we dichotomized the expression patterns into high and low categories (supplementary material, Table S4) 23, 28. Based on the dichotomized values, we selected 28 ‘simple’ cases, which each represent an associated aberration, to be examined by high‐throughput approaches (Figure 2). We were able to detect a small number of mutations in other known prostate cancer drivers using the Ampliseq Cancer Hot‐spot assay (Ion Torrent) within these 28 patients (supplementary material, Figure S1). *TP53* mutation is among the highest in incidence of mutated genes in prostate cancer, with a mutational burden predominantly observed in advanced/metastatic cases. In our cohort, three patients carried *TP53* mutations and these were identified in either PTEN‐L (2 patients) or PTEN‐M (1 patient) but not in PTEN‐H cases. The overall incidence of *TP53* mutations in our cohort (∼10%) was therefore in line with other published studies 29, 30. Because the mutational incidence was low overall, these data were not incorporated into the downstream analysis workflows.

### Distinct gene expression profile associated with *PTEN* expression

Expression of the tumour suppressor *PTEN* gene is reduced in 50–70% of all prostate cancer cases based on transcript profiling 31, 32, 33. More recently PTEN downregulation as assessed by IHC has been reported in 40% of advanced cases and is strongly associated with reduced median survival. 34 We therefore chose to assess PTEN expression by IHC as one potential regulator of cancer progression in grade 7 disease. Loss of PTEN expression at the protein level was observed in 51% of our cohort when assessed by immunohistochemistry (Figure 2). This was further validated at the mRNA level by the whole genome gene expression assay as well as by single marker real‐time PCR analysis, with observed high concordance between the assays (supplementary material, Figure S2 and S4). Interestingly, while we observed differential protein expression for the other six drivers as assessed by IHC, these changes in marker expression did not correlate with concordant changes in their transcript expression or with an associated methylation pattern.

**Figure 3:**
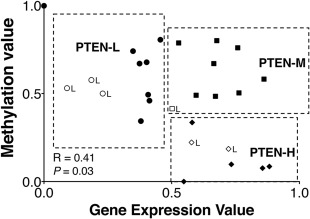
Integrated analysis of untreated Gleason 7 (3 + 4) prostate tumours revealed distinct subgroups associated with PTEN status. Bivariate correlation analysis of whole genome gene expression and methylation data showed the stratification of three subgroups of cases associated with their PTEN status; PTEN‐H (high PTEN gene expression and low methylation); PTEN‐M (high PTEN gene expression and methylation; PTEN‐L (low PTEN gene expression and high methylation). A positive correlation between gene expression and methylation status was observed (*R* = 0.41, *p* = 0.03). Cases with low PTEN immunohistochemistry expression status are denoted with ‘L’ next to each sample case in the correlation plot.

**Figure 4:**
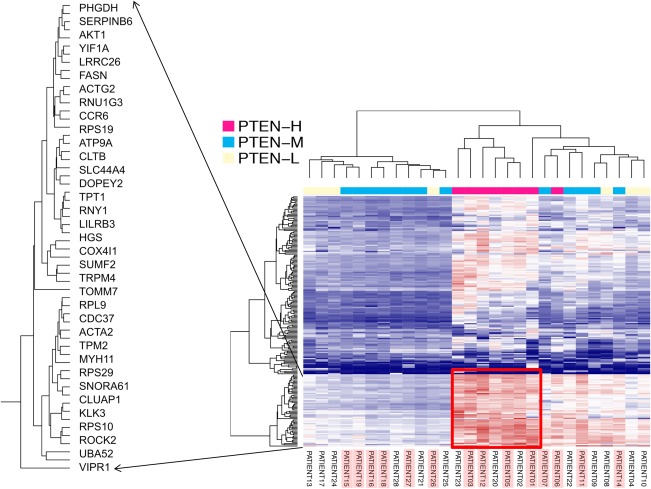
Unsupervised hierarchical clustering of whole genome gene expression data showing clustering of patient subgroups associated with PTEN subtypes identified by bivariate analysis. Patient numbers highlighted with the red box are PTEN protein expressing cases (by immunohistochemistry).

We did not observe any identifiable *PTEN* somatic point mutations through high throughput gene‐targeted sequencing (supplementary material, Figure S1), leading us to hypothesize that there could be distinguishable subgroups of patients with differing *PTEN* status that is based on the level of gene expression and methylation status of *PTEN*. To validate this, we performed a bivariate correlation analysis between the gene expression and the methylation values based on the beta‐values (ranging from 0 to 1). The bivariate correlation analysis showed distinct stratification of the cases into high PTEN expressing (PTEN‐H), moderate PTEN expressing (PTEN‐M), and low PTEN expressing (PTEN‐L) subgroups (*p* < 0.01) (Figure 3). We then sought to investigate if these subtypes can be recapitulated by cluster analysis. By performing unsupervised hierarchical clustering analysis, the separation of PTEN‐H cases from the PTEN‐M and PTEN‐L cases was reiterated. Through cluster analysis, we also observed a distinct expression profile that distinguished the PTEN‐H cases from the other two subtypes (Figure 4).

### Identification of a 35‐gene expression signature associated with prognostic outcome in prostate cancers

From the cluster analysis of the gene expression data, we identified a group of 35 genes that was differentially expressed in PTEN‐H compared to PTEN‐M and PTEN‐L patients in the Northern Ireland gene expression dataset. We sought to investigate the prognostic potential of this signature in defining indolent disease in further cohorts of Gleason 3 + 4 intermediate risk cases. To do this, we tested the prognostic ability of the signature in three published prostate cancer gene expression datasets 20, 21, 22. All three datasets contain extensive clinico‐pathological features, as well as time to recurrence and time to death.

After retrieving the relevant clinical and pathological information, we further limited the survival analysis to Gleason 7 (3 + 4) cases in each dataset. The purpose was to eliminate the possibility of Gleason score being a confounding factor and, furthermore, to align the clinical features in accordance with the features of the Northern Ireland dataset.

**Figure 5:**
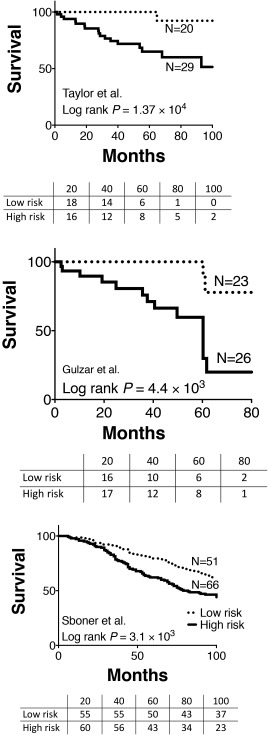
The prognostic value of the 35‐gene expression signature was analysed in three independently published datasets, GSE21032, GSE16560 and GSE40272 [20–22]. The survival plots depicted here were derived from Kaplan–Meier analysis (the *P* values summarize the differences by the log‐rank tests).

We used a risk estimation index based on Cox proportional hazard modelling to determine the prognostic effect of the 35‐gene expression signature. We observed strong survival discrimination in the Taylor (HR, 6.95; 95% CI, 2.73–17.54; *P* = 5.97 × 10^−5^) and Gulzar datasets (HR, 6.40; 95% CI, 2.28–17.96; *P* = 4.21 × 10^−4^). To a lower extent, a similar effect was also observed in the Sboner dataset (HR, 1.77; 95% CI, 1.29–2.41; *P* = 3.25 × 10^−4^). We also observed significant differences in survival based on Kaplan–Meier analysis (Figure 5). We also assessed the individual prognostic power of each gene within the signature and found that 4 of the 45 genes were able individually to provide a HR of 3.21 or greater with *P* value of <0.05 (supplementary material, Table S5).

## Discussion

Loss of *PTEN* is widely recognized as a common genomic aberration in prostate cancer. Generally, it has been suggested that PTEN loss occurs more frequently in metastatic prostate cancers than in primary tumours 20, 35. PTEN loss has previously been detected in intermediate risk disease and has been shown by immunohistochemistry to correlate with recurrence/disease progression. The frequency of loss of PTEN protein expression examined by immunohistochemistry in our study of intermediate cases (51%) is therefore consistent with reported findings 20, 36. There are however currently no markers that associate with PTEN expression and identify patients that will benefit from active surveillance as opposed to treatment.

Our purpose in this study was to identify molecular correlates with PTEN expression and evaluate their prognostic significance. Through cluster analysis, we have derived a distinct gene expression profile that defines a subgroup of PTEN expressing cases. These PTEN‐H cases were marked by the differential expression of a panel of 35 genes that discriminate them from the other two subtypes (PTEN‐M and PTEN‐L). This geneset profile shows significant prognostic effect across three independent datasets that we interrogated (HR of 6.95, 6.4, and 1.77, all *p* < 0.01) (Figure 5 and supplementary material, Figure S5).

**Figure 6:**
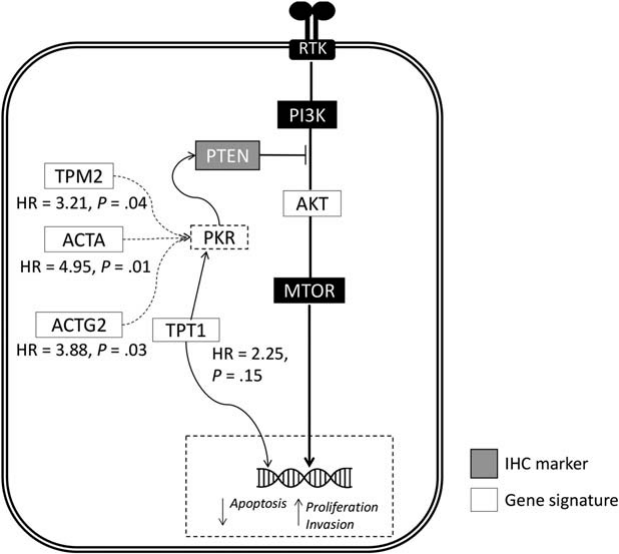
Schematic diagram summarising the biological processes underlying the association of the 35‐gene expression signature with PTEN/AKT signalling pathways. Notably, PKR has been reported to be important for the tumour suppressive activity of PTEN, independent of the PI3K/AKT signalling pathway 42. We hypothesized that this could be modulated by several genes within our gene signature. TPT1 has been previously reported to modulate the expression of PKR 40. Apart from *TPT1*, actins and troponin‐associated factors from the 35‐gene expression signature (*TPM2, ACTA2,* and *ACTG2*) were also reported to induce the activation of *PKR*. The dotted lines in the diagram thus signify biological associations between genes within our signature and its possible association with PKR that warrant further investigation.

The relationship of these genes to the known principal drivers of prostate cancer can be surmised. Assessing a publicly available dataset 37, we have determined that only four of the selected genes are related to androgen receptor activity; FASN, KLK3, TRPM4, and VIPR1 (supplementary material, Figure S3). FASN has previously been reported to be a driver of prostate cancer progression. Analysis of FASN and VIPR1 individually determined that overexpression of these genes yields HRs of 0.81 and 0.70, respectively, with a negative correlation with outcome. Consequently, we ruled out AR activity as a dominant driving biology for this signature. Subsequent analysis focused on the biology of PTEN and, more specifically, the activation of PTEN (supplementary material, Figure S6). PTEN activity is known to be regulated in part by phosphorylation. One key kinase that phosphorylates and activates PTEN is PKR. PKR is itself activated by high levels of expression of a subset of mRNAs carrying a hairpin‐loop secondary structure. Within our 35‐gene PTEN‐H signature, there are also several components that suggest a possible role for PKR activation in mediating PTEN expression, particularly the influence of TPT1. The *TPT1* gene has been previously shown to be associated with disease progression in prostate and colorectal cancers 38, 39. Functionally, TPT1 was reported to be involved in several biological processes, including rapamycin signalling as well as mitosis and nuclear reprogramming 40, 41. Furthermore, gene silencing and knockdown of *TPT1* was reported to inhibit cell proliferation and invasion 38, 39. Notably, it is postulated that the activation of TPT1 modulates the activity of serine‐threonine kinase PKR 40. Mounir and colleagues have previously established the role of PKR in the tumour suppressive activity of PTEN as an alternative link that is independent of the PI3K signalling pathway 42. In their study, they reported that the activation of PKR resulted in the phosphorylation of EIF2 leading to subsequent inhibition of cell proliferation 42. Apart from TPT1, actins and tropomycin have also been described to induce the *in‐vitro* activation of PKR 43. Interestingly, within our PTEN‐H signature, there are several representative factors present, namely TPM2, ACTA2, and ACTG2. These genes individually have high prognostic effect (significant hazard risk ratios, Figure 6). Therefore, we propose that the strong association of genes that are known PKR activators suggests that our signature of good outcome is associated biologically with the retention of PTEN activity and thus the capacity to constrain or retard the rate of tumour progression. PTEN activity assessments in restricted sampling of formalin‐fixed material was beyond the technical and feasible capability of the field and current study design but will be an important aspect of future validation of this signature in prospectively sourced samples.

Our investigations yield a gene signature that is strongly associated with survival outcome in untreated Gleason 7 (3 + 4) prostate cancers that could potentially identify high risks patients who would benefit from early treatment intervention. Currently, biomarkers evaluated in most studies correlate with grade and high‐risk staging, and are thus possibly confounded by tumour proliferation. In our study, we have essentially excluded that bias and proliferative component by limiting the analysis to Gleason 7 (3 + 4) cases. Our signature therefore allows us to further postulate that there is limited prospect of progression for the low risk patients despite pathological presentation of neoplastic phenotypes and overexpression of previously reported prognostic markers such as TPT1. We hypothesize that this is due to the numerous tumour suppressive checkpoints, such as the role of PKR activity. This implies that some of these genes may become ‘oncogenic’ or contribute to aggressive disease once cell cycle checkpoints are compromised. Without such changes, they are but rather regulators of normal energy balance or moderators of stress.

In summary, our study to our knowledge is the first that evaluates markers in a single Gleason score setting using a comprehensive profiling approach. Pending further validation, the 35‐gene expression signature derived from this analysis of untreated Gleason 7 (3 + 4) prostate cancers has the potential to accurately stratify patients and provide enhanced prognostic insights.

## Author contributions statement

DW, CWO, and MST conceived and designed the study. CWO, PM, AA, and SM acquired data. CWO, PM, AA, SM, DW, IM, and MST analysed and interpreted data. CWO drafted the manuscript. DW, IM, and MST revised the manuscript.

## Supporting information

SUPPLEMENTARY MATERIAL ONLINE

**Supplementary figure legends**

**Figure S1.** The number of DNA alterations in the Northern Ireland cohort as identified by the Ion Torrent Ampliseq Cancer Hotspot assay and the corresponding PTEN subtypes and clinical characteristics

**Figure S2.** The scatterplot shows the correlation between RT‐qPCR derived and whole genome DASL‐derived gene expression values for *PTEN* expression (normalized to values of 0‐1). The R value shown is Spearman rho coefficient

**Figure S3.** Time‐based gene expression data from androgen‐stimulated LNCaP cells [37]. Cells were cultured in steroid‐depleted media and treated with 1 nm R1881 over a period of 24 h. These were interrogated to identify AR‐regulated gene members within the 35‐gene expression signature. Four genes (*KLK3, FASN, TRPM4, and VIPR1*) were identified to be highly regulated by stimulation of the androgen receptor (*p < 0.01*)

**Figure S4.** The correlation between immunohistochemistry and (A) gene expression, (B) methylation, and (C) RT‐qPCR (unpaired t‐test)

**Figure S5**. ROC analysis of the prognostic potential of the 35‐gene signature (AUC 0.719) in the validation cohort (Taylor, Gulzar, and Sboner) compared to PTEN as a single marker (AUC 0.567)

**Figure S6**. *PTEN* gene expression by risk status in the validation cohort (Taylor, Gulzar and Sboner)

**Table S1.** The clinical characteristics of the retrospective Northern Ireland patient cohort
**Table S2.** Description of the prostate cancer datasets used in this study and their respective institutions
**Table S3.** Immunohistochemistry conditions and antibodies used in this study
**Table S4.** Table showing the definition of the expression score obtained for each TMA core based on Allred scoring criteria [23]
**Table S5.** The individual hazard risk ratios (HR) of each gene from the 35‐gene signature in the Taylor's cohort[20]
