# Supplementary material for: A gene signature associated with PTEN activation defines good prognosis intermediate risk prostate cancer cases
Source: J Pathol Clin Res. 2018 Feb 28;4(2):103–13. doi: 10.1002/cjp2.94 (PMC5903700; doi:10.1002/cjp2.94)
Supplement: Supplementary file 1 — Supplementary figure legends [file CJP2-4-103-s007.docx]

**Supplementary Figure Legends**

**Figure S1.** The number of DNA alterations in the Northern Ireland cohort as identified by the Ion Torrent Ampliseq Cancer Hotspot assay and the corresponding PTEN subtypes and clinical characteristics.

**Figure S2.** The scatterplot shows the correlation between RT-qPCR derived and whole genome DASL-derived gene expression values for *PTEN* expression (normalized to values of 0–1). The *R* value shown is Spearman rho coefficient.

**Figure S3.** Time-based gene expression data from androgen-stimulated LNCaP cells [37]. Cells were cultured in steroid-depleted media and treated with 1 nm R1881 over a period of 24 h. These were interrogated to identify AR-regulated gene members within the 35-gene expression signature. Four genes (*KLK3*, *FASN*, *TRPM4,* and *VIPR1*) were identified to be highly regulated by stimulation of the androgen receptor (*p* < 0.01).

**Figure S4.** The correlation between immunohistochemistry and (A) gene expression, (B) methylation, and (C) RT-qPCR (unpaired *t*-test).

**Figure S5**. ROC analysis of the prognostic potential of the 35-gene signature (AUC 0.719) in the validation cohort (Taylor, Gulzar, and Sboner) compared to PTEN as a single marker (AUC 0.567).

**Figure S6**. *PTEN* gene expression by risk status in the validation cohort (Taylor, Gulzar, and Sboner).
