# Supplementary figures and images for: A gene signature associated with PTEN activation defines good prognosis intermediate risk prostate cancer cases
Source: J Pathol Clin Res. 2018 Feb 28;4(2):103–13. doi: 10.1002/cjp2.94 (PMC5903700; doi:10.1002/cjp2.94)

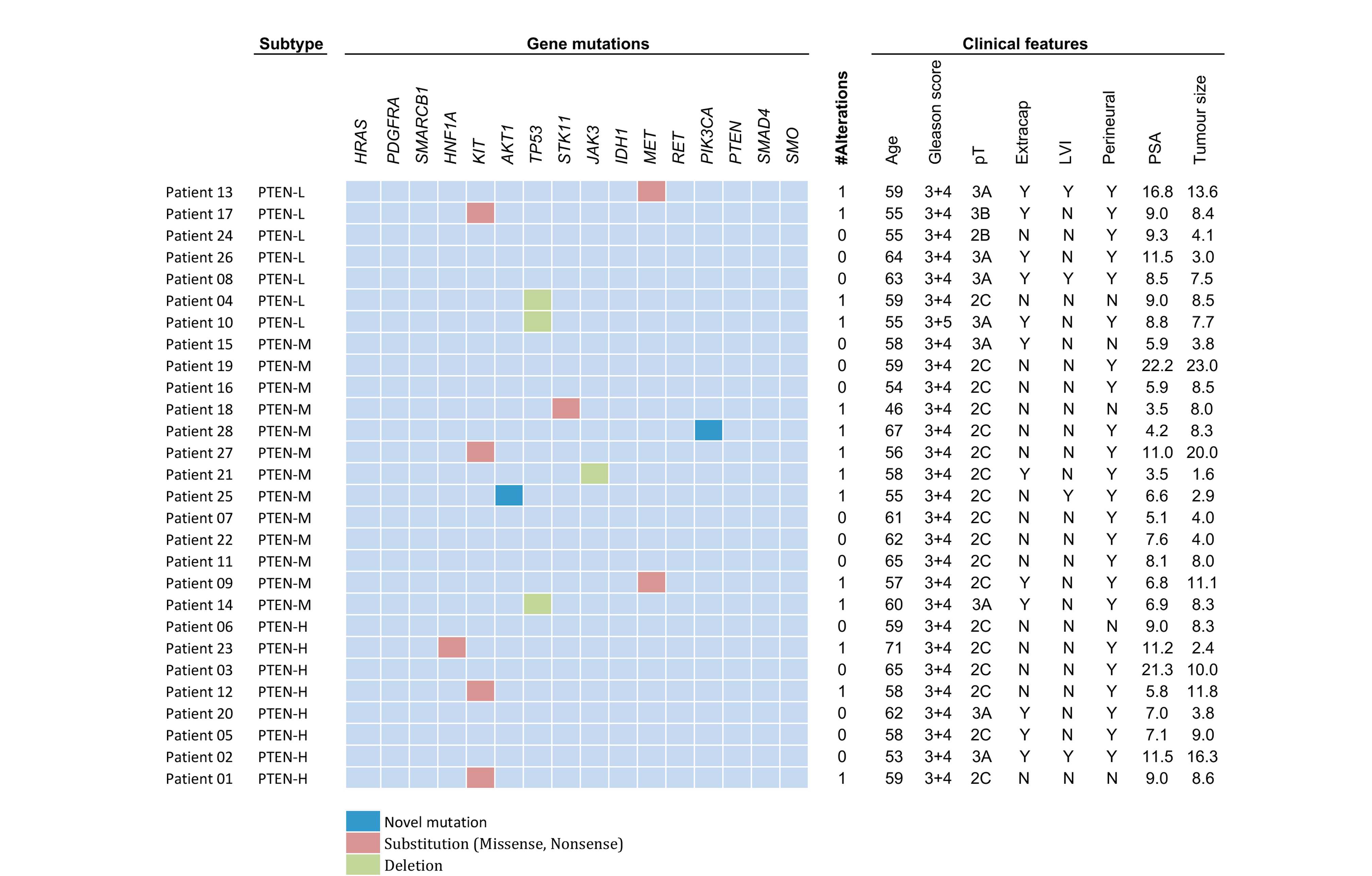

Supplement: Supplementary file 2 — Figure S1. The number of DNA alterations in the Northern Ireland cohort as identified by the Ion Torrent Ampliseq Cancer Hotspot assay and the corresponding PTEN subtypes and clinical characteristics [file CJP2-4-103-s001.tif]

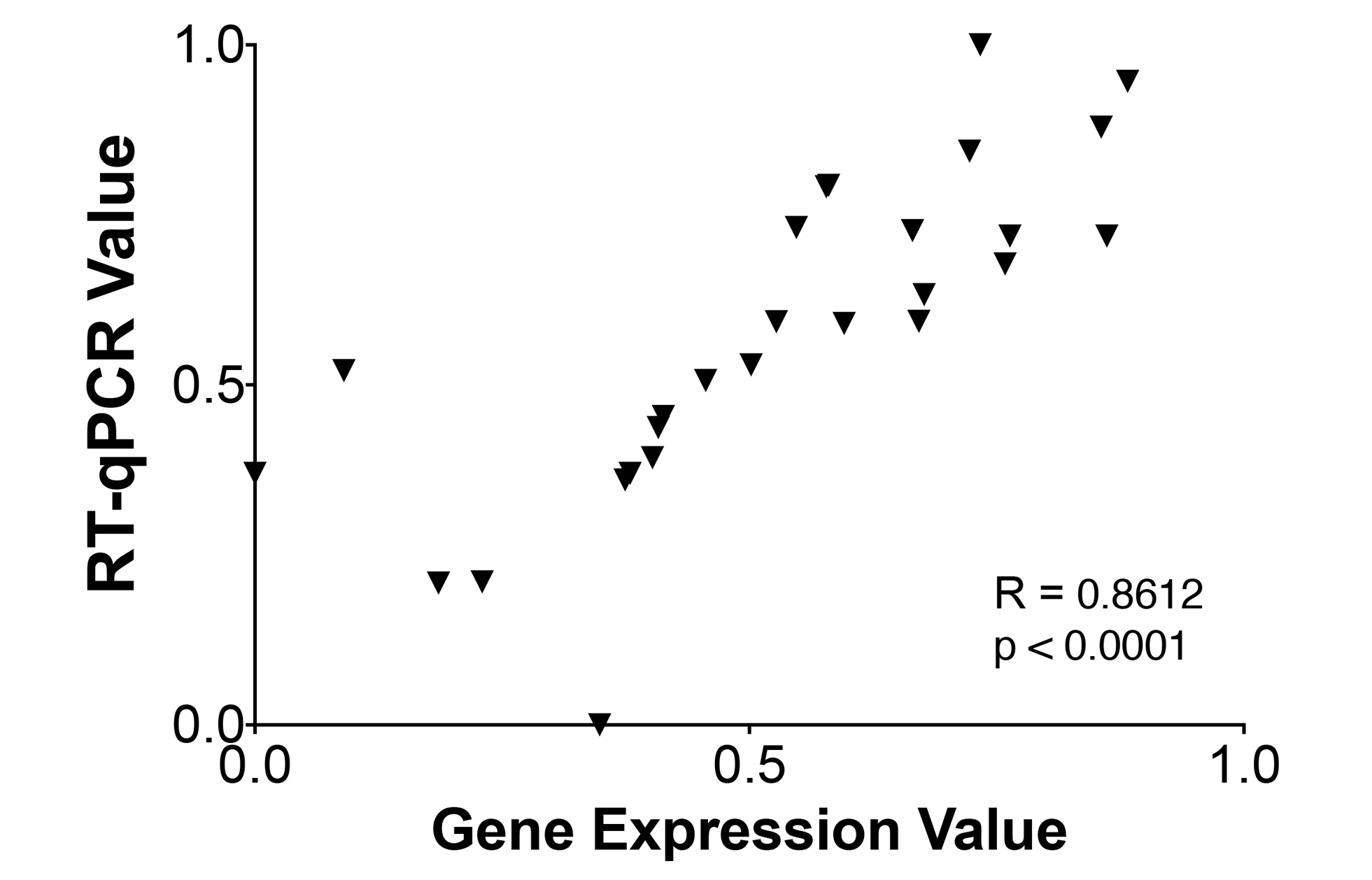

Supplement: Supplementary file 3 — Figure S2. The scatterplot shows the correlation between RT‐qPCR derived and whole genome DASL‐derived gene expression values for PTEN expression (normalized to values of 0‐1). The R value shown is Spearman rho coefficient [file CJP2-4-103-s002.tif]

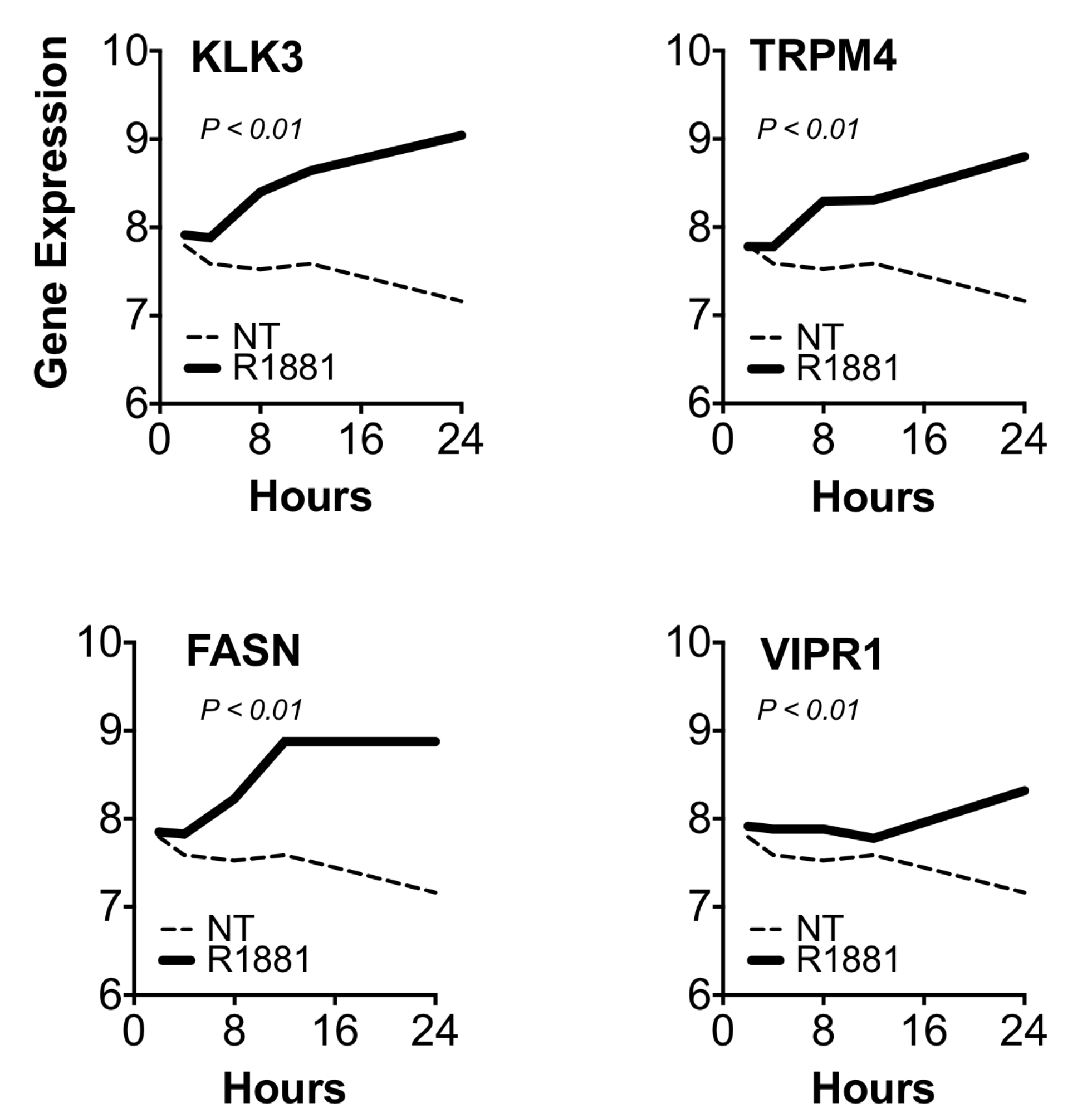

Supplement: Supplementary file 4 — Figure S3. Time‐based gene expression data from androgen‐stimulated LNCaP cells [37]. Cells were cultured in steroid‐depleted media and treated with 1 nm R1881 over a period of 24 h. These were interrogated to identify AR‐regulated gene members within the 35‐gene expression signature. Four genes (KLK3, FASN, TRPM4, and VIPR1) were identified to be highly regulated by stimulation of the androgen receptor (p < 0.01) [file CJP2-4-103-s003.tif]

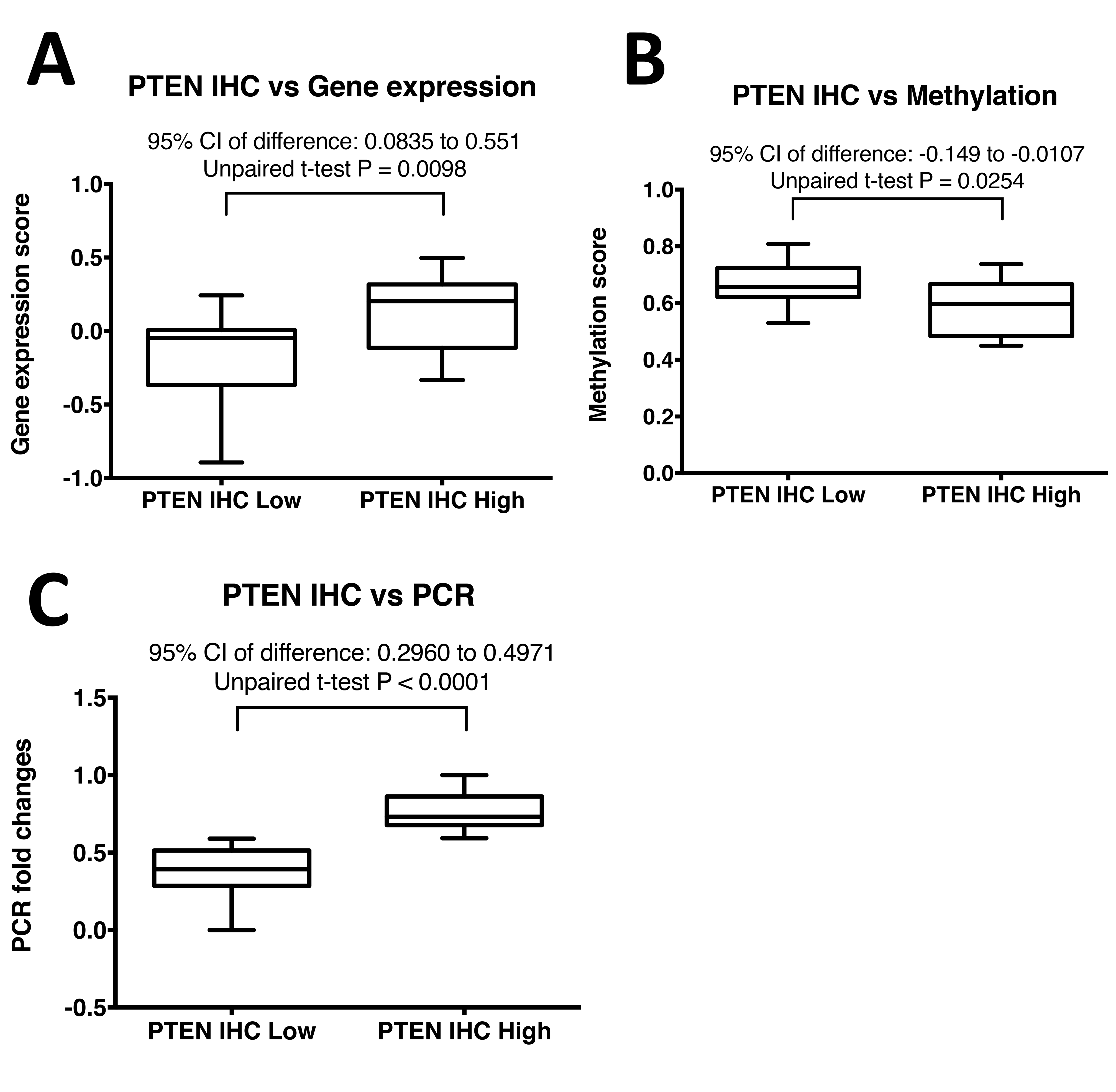

Supplement: Supplementary file 5 — Figure S4. The correlation between immunohistochemistry and (A) gene expression, (B) methylation, and (C) RT‐qPCR (unpaired t‐test) [file CJP2-4-103-s004.tif]

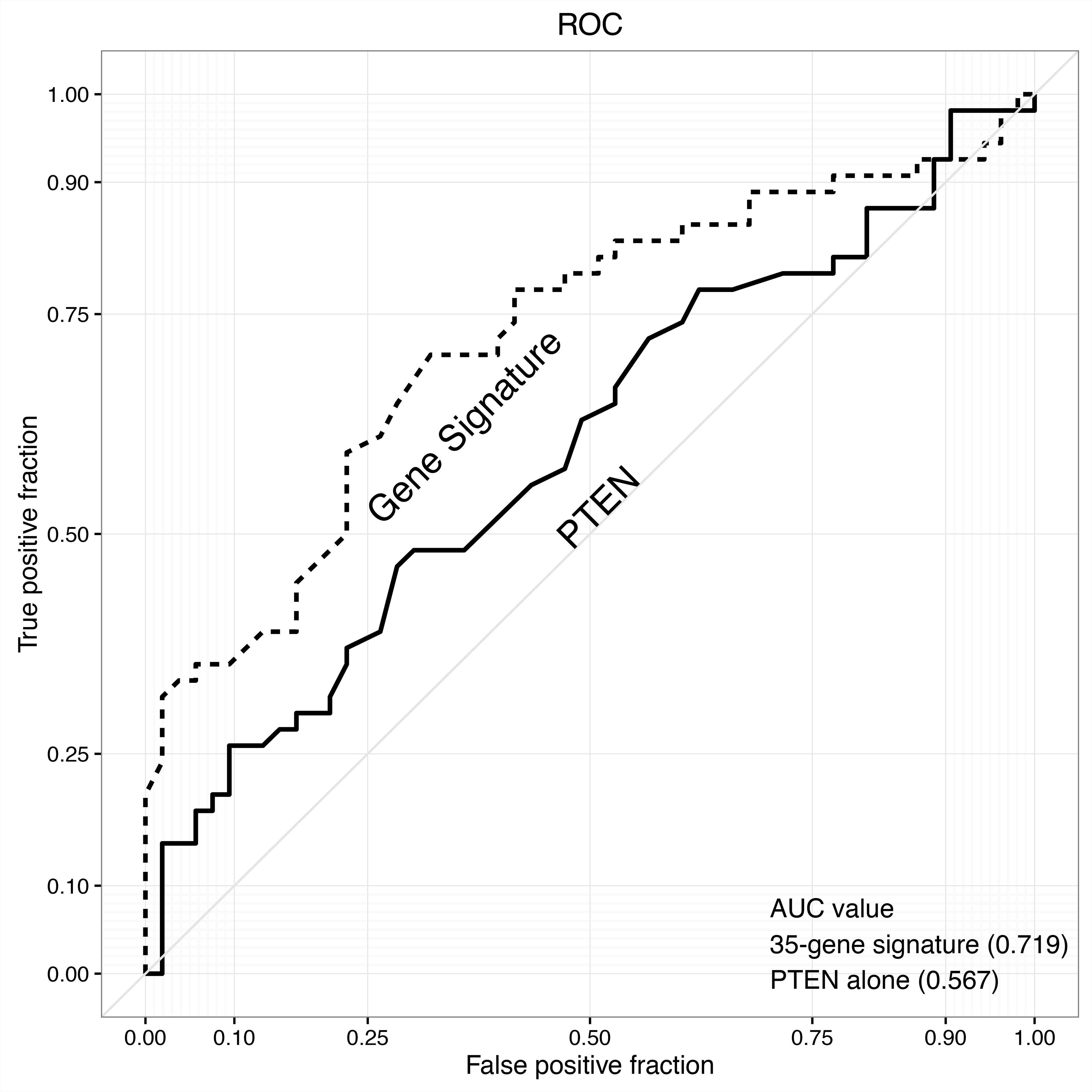

Supplement: Supplementary file 6 — Figure S5. ROC analysis of the prognostic potential of the 35‐gene signature (AUC 0.719) in the validation cohort (Taylor, Gulzar, and Sboner) compared to PTEN as a single marker (AUC 0.567) [file CJP2-4-103-s005.tif]

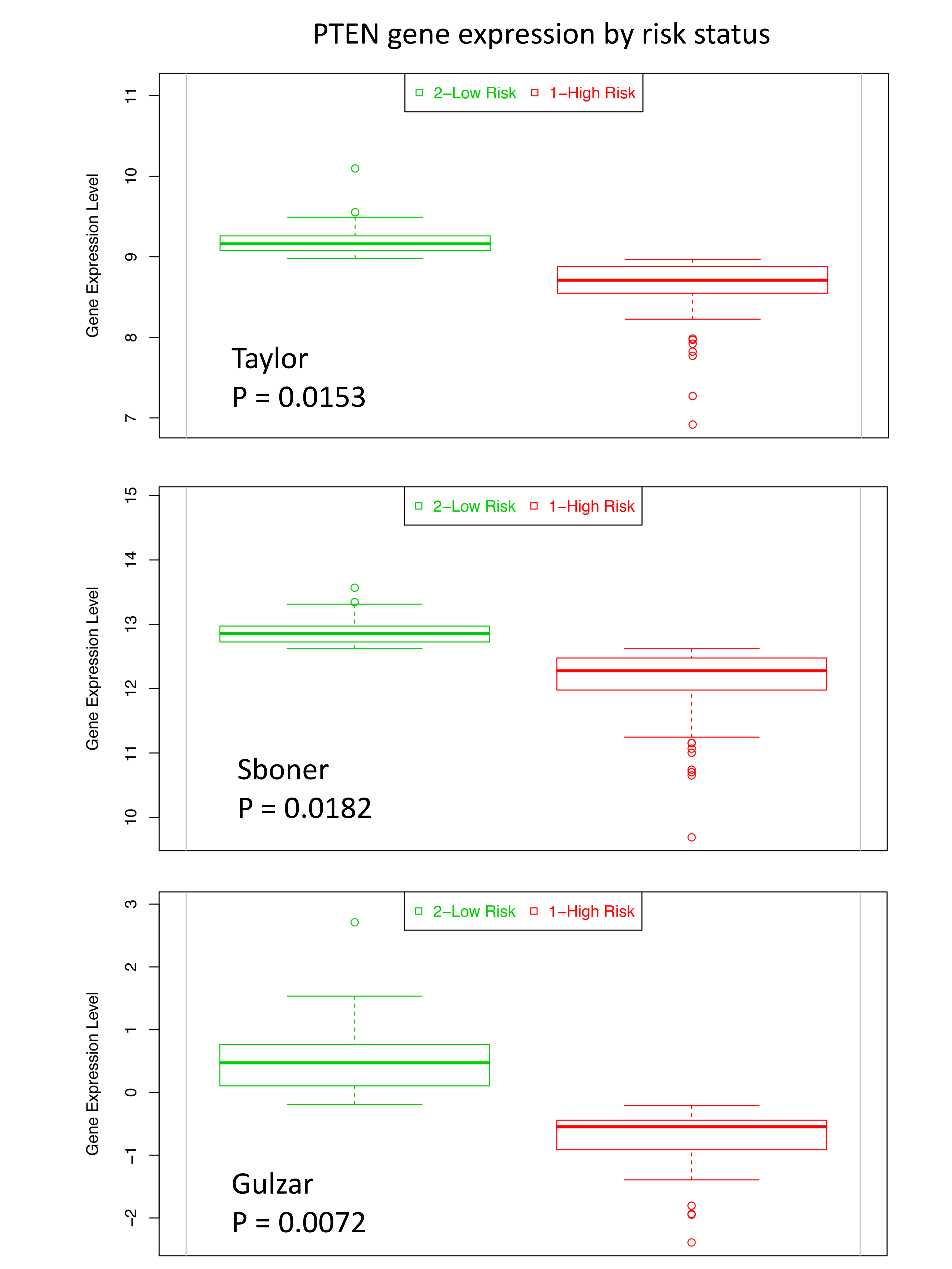

Supplement: Supplementary file 7 — Figure S6. PTEN gene expression by risk status in the validation cohort (Taylor, Gulzar and Sboner) [file CJP2-4-103-s006.tif]
