# Supplementary material for: A gene signature associated with PTEN activation defines good prognosis intermediate risk prostate cancer cases
Source: J Pathol Clin Res. 2018 Feb 28;4(2):103–13. doi: 10.1002/cjp2.94 (PMC5903700; doi:10.1002/cjp2.94)
Supplement: Supplementary file 8 — Table S1. The clinical characteristics of the retrospective Northern Ireland patient cohort Table S2. Description of the prostate cancer datasets used in this study and their respective institutions Table S3. Immunohistochemistry conditions and antibodies used in this study Table S4. Table showing the definition of the expression score obtained for each TMA core based on Allred scoring criteria [23] Table S5. The individual hazard risk ratios (HR) of each gene from the 35‐gene signature in the Taylor's cohort[20] [file CJP2-4-103-s008.docx]

**Supplementary Tables**

**Table S1.** The clinical characteristics of the retrospective Northern Ireland patient cohort

| **Characteristic** | **Northern Ireland cohort (N = 62)** | **Percentage** |
| --- | --- | --- |
| Surgery year, range | 2009–2013 |  |
| Age, mean years | 58.34 |  |
| Gleason score |  |  |
| *≤ 6* | 5 | 8.1 |
| *7 (3 + 4)* | 55 | 88.7 |
| *≥ 8* | 2 | 3.2 |
| Clinical staging | | |
| *T2* | 37 | 59.7 |
| *T3* | 25 | 40.3 |
| Baseline PSA level | | |
| *≤ 4* | 5 | 8.1 |
| *> 4–10* | 39 | 62.9 |
| *>10–20* | 10 | 16.1 |
| *>20* | 2 | 3.2 |
| *No available information* | 6 | 9.7 |

PSA, prostate-specific antigen.

Table S2. Description of the prostate cancer datasets used in this study and their respective institutions

| **Reference** | **Institution** | **Sample size (Gleason 3 + 4)** | **Sample type** | **Outcome measures** | **Database** |
| --- | --- | --- | --- | --- | --- |
| Taylor et al. [20] | Memorial Sloan-Kettering Cancer Center | 66 | RP | RFS | GSE21032 |
| Sboner et al. [21] | Weill Cornell Medical College | 281 | TURP | OS | GSE16560 |
| Gulzar et al. [22] | Stanford University | 98 | RP | RFS | GSE40272 |

**Table S3.** Immunohistochemistry conditions and antibodies used in this study

| **Protein** | **Gene description** | **Clone** | **Manufacturer** | **Conditions** |
| --- | --- | --- | --- | --- |
| AR | Androgen receptor | AR441 | Abcam | 1:200 |
| AURKA | Aurora kinase A | 35C1 | Abcam | 1:100 |
| ERG | V-ets avian erythroblastosis virus E26 oncogene homolog | EPR3864 | Abcam | 1:250 |
| MYC | V-myc avian myelocytomatosis viral oncogene homolog | 9E10 | Abcam | 1:500 |
| RB1 | Retinoblastoma 1 | G3-245 | BD Pharmingen | 1:200 |
| PTEN | Phosphatase and tensin homolog | 6H2.1 | Dako | 1:1600 |
| TP53 | Tumour protein 53 | DO-7 | Dako | 1:100 |

Table S4. Table showing the definition of the expression score obtained for each TMA core based on Allred scoring criteria [23]

| **Intensity score** | **Proportion of positively stained cells (%)** | **Expression score** |
| --- | --- | --- |
| 0 (negative) | 0% | Low expressing |
| 1+ (weak) | Any proportion | Low expressing |
| 2+ (moderate) | < 50% | Low expressing |
| 2+ (moderate) | ≥ 50% | High expressing |
| 3+ (strong) | Any proportion | High expressing |

**Table S5.** The individual hazard risk ratios (HR) of each gene from the 35-gene signature in the Taylor’s cohort [20]

| **Gene** | **HR (95% CI)** | ***p*** |
| --- | --- | --- |
| ACTA2 | 4.95 (1.41–17.34) | 0.0125 |
| ACTG2 | 3.88 (1.11–13.56) | 0.0335 |
| MYH11 | 3.35 (1.09–10.31) | 0.0347 |
| TPM2 | 3.21 (1.04–9.92) | 0.0428 |
| ROCK2 | 2.76 (0.91–7.45) | 0.0738 |
| COX4I1 | 2.33 (0.86–6.33) | 0.095 |
| UBA52 | 2.27 (0.63–8.09) | 0.2076 |
| TPT1 | 2.26 (0.74–6.95) | 0.1531 |
| TRPM4 | 1.99 (0.70–5.66) | 0.1974 |
| DOPEY2 | 1.58 [0.86–2.89] | 0.4672 |
| RNY1 | 1.40 (0.52–3.80) | 0.5085 |
| CLTB | 1.37 [0.90–2.08] | 0.1408 |
| TOMM7 | 1.35 [0.89–2.03] | 0.3596 |
| KLK3 | 1.32 [0.23–7.47] | 0.011 |
| RPS10 | 1.32 [0.23–7.47] | 0.2698 |
| LRRC26 | 1.26 [0.83 - 1.91] | 0.7323 |
| ATP9A | 1.18 [0.87–1.62] | 0.2287 |
| CDC37 | 1.18 [0.77–1.83] | 0.4504 |
| SUMF2 | 1.16 (0.41–3.29) | 0.7813 |
| SNORA61 | 1.15 [0.81–1.64] | 0.3427 |
| CLUAP1 | 1.13 (0.43–2.97) | 0.8083 |
| PHGDH | 1.12 [0.93–1.35] | 0.2324 |
| SLC44A4 | 1.12 [0.71–1.79] | 0.1528 |
| YIF1A | 1.05 [0.87–1.27]] | 0.2391 |
| AKT1 | 1.02 [0.86–1.21] | 0.2641 |
| RPL9 | 1.01 [0.81–1.26] | 0.2242 |
| RPS29 | 1.01 [0.75–1.37] | 0.4233 |
| RNU1G3 | 1.01 (0.39–2.63) | 0.9794 |
| LILRB3 | 0.98 (0.37–2.59) | 0.9681 |
| HGS | 0.97 [0.78–1.19] | 0.2759 |
| SERPINB6 | 0.97 [0.43–2.19] | 0.9427 |
| CCR6 | 0.93 [0.77–1.12] | 0.4504 |
| RPS19 | 0.82 [0.64–1.06] | 0.1306 |
| FASN | 0.81 [0.56–1.16] | 0.2547 |
| VIPR1 | 0.70 [0.56–0.87] | 0.0013 |

The genes were ranked in descending order based on their HR values.
